# The right of access to healthcare: an analysis of how legal and institutional frameworks constrain or facilitate access to healthcare for residents in border areas in the East African Community

**DOI:** 10.1186/s12939-022-01785-3

**Published:** 2022-11-26

**Authors:** Freddie Ssengooba, Susan Babirye, Doreen Tuhebwe, Aloysius Ssennyonjo, Steven Ssendagire, Arthur Rutaroh, Leon Mutesa, Mabel Nangami

**Affiliations:** 1 Department of Health Policy Planning and Management, School of Public Health, Makerere University, Kampala, Uganda; 2 Health Economics and Policy, African Health Economics and Policy Association, Kampala, Uganda; 3 College of Medicine and Health Sciences, University of Rwanda, Kigali, Rwanda; 4 Department of Health Policy and Management, School of Public Health, College of Health Sciences, Moi University, Eldoret, Kenya

**Keywords:** Cross-border health, Access to healthcare, Border residents, Legal frameworks, EAC, East Africa, Institutional frameworks

## Abstract

**Background:**

Despite many countries working hard to attain Universal Health Coverage (UHC) and the Health-related Sustainable Development Goals, access to healthcare services has remained a challenge for communities residing along national borders in the East Africa Community (EAC). Unlike the communities in the interior, those along national borders are more likely to face access barriers and exclusion due to low health investments and inter-state rules for non-citizens. This study explored the legal and institutional frameworks that facilitate or constrain access to healthcare services for communities residing along the national borders in EAC.

**Methods:**

This study is part of a broader research implemented in East Africa (2018–2020), employing mixed methods. For this paper, we report data from a literature review, key informant interviews and sub-national dialogues with officials involved in planning and implementing health and migration services in EAC. The documents reviewed included regional and national treaties, conventions, policies and access rules, regulations and guidelines that affect border crossing and access to healthcare services. These were retrieved from official online and physical libraries and archives.

**Results:**

Overall, the existing laws, policies and guidelines at all levels do not explicitly deal with cross border healthcare access especially for border residents, but address citizen rights and entitlements including health within national frameworks. There is no clarity on whether these rights can be enjoyed beyond one’s country of citizenship. The review found examples of investments in shared health infrastructure to benefit all EAC member countries – a signal of closer cooperation for specialized health care, this had not been accompanied by access rule for citizens outside the host country. The focus on specialized care is unlikely to contribute to the every-day health care needs of border resident communities in remote areas of EAC. Nevertheless, the establishment of the EAC entail opportunities for increased collaboration and integration beyond the trade and customs union to included health care and other social services. The study established active cooperation aimed at disease surveillance and epidemic control among sub-national officials responsible for health and migration services across borders. Health insurance cards, national identification cards and official travel documents were found to constrain access to health services across the borders in EAC.

**Conclusion:**

In the era of UHC, there is need to take advantage of the EAC integration to revise legal and policy frameworks to leverage existing investments and facilitate cross-border access to healthcare services for communities residing along EAC borders.

**Supplementary Information:**

The online version contains supplementary material available at 10.1186/s12939-022-01785-3.

## Background

The discourse on pandemics like COVID-19, Ebola, and undocumented immigration has heightened the vulnerability of non-citizens in accessing healthcare and the need to extend social protection and essential entitlements for non-citizens. Despite health being a fundamental right, access to cross border health care especially for communities residing along international borders remains a challenge [1]. At the onset of COVID-19 pandemic the standoff created by transnational truck drivers and their COVID-19 testing at boarder points in EAC created serious diplomatic discord at ministerial and heads of state fora. Unfortunately, these standoffs resulted in heavily crowded border areas and aided faster transmission of COVID-19 in those communities [2]. Similar problems were witnessed across United Kingdom (UK)-France and United States of America (USA)-Mexico boarders.

Border areas are usually at the periphery from the country’s cities and towns, mostly marginalized, under-invested and economically deprived – a situation that push “border resident communities” to fringe for health care systems [3]. The problems of health care access across national borders are well known in the developed world. For example, patient migration between Mexico and the USA, and within the European Union are well documented [4, 5]. Medical Tourism is now a big industry – all build to serve the healthcare needed that are more efficiently address from ones country of citizenship. The existence of different state and territorial entitlements to health care services [6] is more likely to affect communities that are more proximal to these territorial borders where access barriers due to administrative, legal and regulatory frameworks become more intense and palpable. The political agenda to build a wall between Texas and Mexico as espoused by the Trump presidency (2016–2020) [7] is an example of the physical barriers that limits the cross-border health care for affected communities. Other cross-border barriers may relate to disparities in healthcare investments, quality of healthcare, differences in referral system, treatment protocols and regulatory standards and practices [1, 8].

Evidence from the European Union – where the context of political integration prevails, highlights several reasons for patient mobility in border-regions for member states. As observed by Glinos and Baeten (2006) patient mobility is often due to the physical nature of the borders; cross-border distances are often shorter than going elsewhere within one’s own country of residence; people are likely to be familiar with health care facilities ‘on the other side’; and crossing the border is not necessarily felt like going abroad [5]. Other reasons for patient mobility in border-regions include; familiarity with language spoken ‘on the other side’ or even sharing the same dialect, availability in terms of quantity and type of services, variations in financial costs and perceived quality and cultural closeness to the next-door border-region than to nearby regions in one’s own country [7].

In USA, there are border insurance policies available that cover the care that border residents receive in Mexico and this provides incentives to access the less costly care across the border [3].

In contexts where cross border health has advanced, it has been facilitated by legislation. For example, the cross-border healthcare directive was a major step forward for ensuring patients access to safe and high-quality healthcare across national borders in the European Union [8]. Likewise, the Treaty for the Establishment of the East African Community provides for the promotion of the management of health delivery systems and better planning mechanisms to enhance efficiency within partner states. Despite that, minimal strides have been made in the development of policies to enhance cooperation across health systems of member states. These legal frameworks functions as social and political determinants of health more broadly by defining the parameters of people’s entitlements, the responsibilities of the different levels of government and regulation of players. In general, there is a dearth of basic information and statistics about health and development for East Africa communities residing in border-regions. Health policies targeting these communities and concern among policy maker are often lacking or non-existent. WHO also acknowledges that as national borders become increasingly porous, public health experts need to explore new ways of managing national health systems [9]. This calls for an understanding of the existing legal frameworks in order to harmonize conflicting or unclear laws/policies and formulate favorable policies for promoting cross-border health access.

This paper aims to explore the current legal and policy frameworks and how these facilitate or constrain access to healthcare services for communities residing along the East Africa Community (EAC) borders in order to guide policy developments to advance cross-border health in the EAC.

## The EAC context

The East African Community is an intergovernmental organization composed of six countries in the African Great Lakes region in East Africa; Uganda, Kenya, Tanzania, Rwanda, Burundi and Southern Sudan. The community aims to aims to widen and deepen co-operation among the partner states and other regional economic communities in political, economic and social fields for their mutual benefit. Established in 2000, EAC is one of the fastest growing regional economic and political blocs in the African Union (AU). AU is a continental union consisting of 55 member states located on the African continent and it supports political and economic integration among its member states. Although the EAC treaty allows cooperation in several areas, EAC policies have mainly prioritized strategies for economic growth i.e. trade integration, enhancement of product markets and value addition for exports [10].

Opportunity exists in the current political integration (federation) discourse for expanding social protection programs including health care. A regional parliament and a functional secretariat now exist in Arusha Tanzania with the mandate to work out the integration agenda for all EAC countries to operationalize the federation agenda including common administrative, political and social-economic frameworks that enable free movement of goods and services. Although the discourse and efforts of the EAC have initially concentrated on economic and political integration, attention is slowly moving to the social aspects of member states. For instance, the vision for health-related cooperation in the EAC is now in place - *“Undertaking joint action towards the prevention and control of communicable and non-communicable diseases and to control pandemics and epidemics of communicable and vector-borne diseases that might endanger the health and welfare of the residents of the Community and cooperating in facilitating mass immunization and other public health community campaigns*” [11].

## Methods

### Study design

The study utilized a qualitative research design using document review, Key informant (KI) interviews, and dialogue meetings conducted between May 2018 and February 2019. The study was conducted in Uganda, Rwanda, Kenya and Tanzania. The four countries were selected based on their geographical connectedness and unique healthcare system features.

### Document review

This step involved a review of international/regional treaties, conventions, country specific laws, regulations/guidelines/ key legislative acts and policies in order to gather data on existing laws, policies, guidelines and structures that support/hinder cross-border health access in East Africa. We extracted *clauses or specific texts* that had implication for border crossing and access to healthcare services among communities residing in border regions of East Africa. The East Africa Countries targeted included Uganda, Kenya, Tanzania and Rwanda.

Documents were identified and obtained from a pragmatic and purposive search of international and national electronic databases of treaties/conventions, and websites of the East African government entities and professional bodies. We started off with a search for strategic documents including the universal declaration of human rights, country specific constitutions, national development plans, visions for the health care sector, health policies among others (see Table 1).

**Table 1:** Documents alluding to health care access at all levels

Level	Type of documents	Number of documents Reviewed
International level	Treaties/Convention/Covenants/ Declaration/Resolutions/ Charters	8
Regional	Treaties/protocols/conventions, Regional Plans and Policies	9
National level	National Strategies, National constitutions, National Charters, National policies and National Frameworks	46

The electronic database search yielded 12 documents for Uganda, 13 for Kenya, 9 for Tanzania, 12 for Rwanda, 9 for East Africa Community (Regional Documents) and 8 for International treaties and conventions. Of all documents 63 were considered relevant for this study. We used abstraction form to capture particulars of the document, and clauses/specifications. The parameters that guided the literature search and review are rights, entitlements, obligations and institutional arrangements for cross-bordar access to health care for non-residents (Table 2).

**Table 2:** Definition of the parameters that guided the literature search and review

Parameter	Definition
Rights	These are freedom or autonomy for healthcare access by citizens and/ or non-citizens across the borders [12]
Entitlements	These are privileges or benefits granted to citizens and/ or non-citizens by law [13]
Obligations	These are responsibilities or duties for authorities, citizens and/or non-citizens to provide or seek healthcare [14]
Institutional arrangements	These are formal or informal regulations, systems, and processes linked to health care access by citizens and/or non-citizens [15]

The data extraction tools were operationalized to pick the needed information and abstraction was done by a multidisciplinary team. Two independent researchers reviewed each document in order not to miss out any clauses and later built consensus around the extracted clauses. The review focused on healthcare access within border regions and not on other topics such as professional mobility not seeking work opportunities or receive training across the border.

### Key informant interviews

This study also involved policy makers at regional level as key informants to the status of implementing the EAC integration and specifically health integration. The policy makers included two technical representatives from East Central and Southern Africa Health Community (ECSA) Head Quarter, two representatives from the EAC secretariat, and two representatives at the East Africa Parliament. An interview guide for the Key Informant interviews was to gather information on the current legal and policy frameworks as well as practices that facilitated healthcare access across the border in the EAC. The guide was in English and the interviews were conducted in English – an official language for the EAC. The interview guide explored the existence of laws, policies, and guidelines that support and cross-border health access in East Africa; and other arrangements that promote, protect and fulfill health access rights for people residing in cross-border regions of East Africa. The guide included questions such as; (1) at regional or country level, what guidelines are there for allowing or not allowing people to cross and use health services in the neighboring countries; and (2) under what situations, do persons from the opposite side of the national borders receive health care services for treatment such as HIV, maternal delivery and routine vaccination.

Six interviews with policy makers at regional level (East Africa) were undertaken. The KIs included four representatives from the East African Community Secretariat, and two from the East Central and Southern Africa Health Community. Interviews were conducted by one of the authors of the paper (AR) and the participants were purposively selected based on their positions, and experience in policy making at national and regional level. Prior to collecting the data, the study team reviewed the interview guide in detail. All interviews were digitally audio recorded and subsequently transcribed verbatim.

### Dialogue meeting with border district officials

Over 100 national and subnational stakeholders were engaged in four different deliberative meetings held at selected borders areas. Each meeting constituted about 20–25 participants, and at the beginning of every meeting, participants were informed about the purpose of the meeting, including intensions to analyze and publish excepts from the meeting deliberations. The stakeholders engaged through these dialogues included officials responsible for health, migration and related program staff in EAC secretariate. The meeting deliberations focused on practices, guiding laws, rules and tools affecting cross-border access to healthcare, and innovations and arrangements that promote, protect and fulfill health access rights for people residing in cross-border regions of East Africa. The guiding questions included; *what institutional arrangements exist at sub-national level that facilitate or limit cross border healthcare access? How can access to healthcare be improved for cross border communities in the EAC?* These broad questions were used to structure and guide the meeting and the contributions of the participants.

The meetings also served as dissemination avenues for the findings from the review of legal and policy frameworks. These findings also contributed to the meeting deliberations. The meetings were conducted in English the official language in East Africa, and the meeting participants were able to dialogue competently in English without the need for translations into Swahili or other dialects.

### Data analysis and synthesis

The data extracted from the various documents (international, regional and national) were collated. Data was later analyzed using thematic content analysis [16]. Research team members reviewed the summary extracts and jointly generated codes to categories the data according to enabling or constraining factors, migration, rights, obligations and entitlements of citizens for health access. The research team held working meetings to discuss the codes and categorize them. By analyzing and sorting codes, the research team identified consistent and overarching themes as well as supporting sub-themes.

The Key Informant interviews and dialogue meetings were also analyzed using thematic content analysis [16]. We developed a coding framework using deductive codes based on the questions used the interview guide and meetings. illustrative quotations for each theme were identified, discussed and prioritized [17].

## Results

Overall, the existing laws, policies and guidelines at all levels do not explicitly deal with cross border healthcare access especially for border residents, but address citizen rights and entitlements including health within national frameworks. Detailed findings are presented based on the predominant themes identified from the documents reviewed, dialogues and the interviews with key informants on the existing laws, policies, guidelines and structures that support or hinder cross-border health access for populations residing in border regions of East Africa. In particular, three themes merged including; (1) rights, entitlements, and obligations; (2) institutional frameworks and arrangements; and (3) opportunities for cross border healthcare access.

### Rights, entitlements, and obligations

Two categories of rights, entitlements and obligations were identified from the documents reviewed, dialogues and the interviews with key informants. These included; institutionalization of the right to health and its restrictions; and implied access to authorizations for cross border health.

### Institutionalization of the right to health and its restrictions

From the documents reviewed, we found that the existing legal and policy frameworks focused on individual countries and were not explicit about the rights, entitlements and obligations related to healthcare access for populations residing in border regions. There was convergence across the laws and policies reviewed regarding institutionalization of the right to health across the East African Countries however, this right to health was limited to only citizens of the respective country and also by the state capacity to provide health services.

**Fig. 1:**
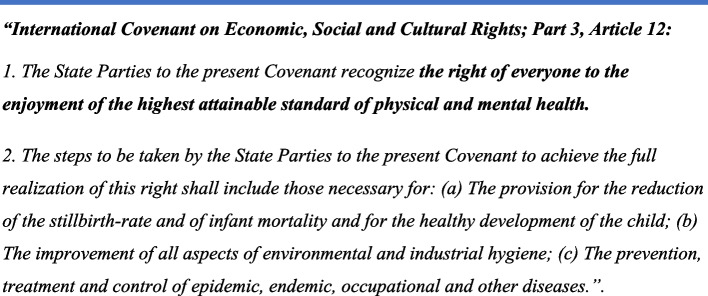
Extract from the International Covenant  on Economic, Social and Cultural rights

For example; the right to health is a universal right established in numerous international and regional human rights treaties, and in all the national constitutions. Specifically, this right is highlighted in the; Universal Declaration of Human Rights (Article 25), International Covenant on Economic, Social and Cultural Rights (ICESCR), (Article 12); International Convention on the Elimination of All Forms of Racial Discrimination (Article 5), International Convention on the Elimination of All Forms of Discrimination Against Women (CEDAW) (Article 12); Convention on the Rights of the Child (CRC) (Article 24). These documents however, also mention the restriction of the right to health to only state citizens (See Fig. 1).

Relatedly, and from the interviews held with the regional policy makers, they also noted the lack of specific policies regarding or guiding access to healthcare across State boundaries in East Africa. The policy makers confirmed that health in the EAC region was still largely guided by policies that were country specific and thus the entitlements to health care services restricted within citizenship;

*“We don’t have a law that formally allows people to move and access health services across borders. Each country currently remains autonomous in handling and budgeting for the health of its citizens” *Policy maker 3, EAC Secretariat.

In the same vein, there was convergence across national level documents in regards to promoting the right to health care access for their country specific citizens. For instance, the Rwanda constitution alludes to the right to health for all Rwandans. These rights were restricted within citizenship and no provisions were made for border communities. Country specific arrangements for healthcare raised implementation issues as highlighted in the dialogues with the officials responsible for health, migration and related program staff at cross border sites. For example;

*The budgeting and service planning were said to be locked to national estimation of need thus not taking cognizance of the services use communities from neighboring country. Dialogue meetings recommended the need to revise policies and guidelines to enable the resource planning and budgets to take care of additional service needs of the communities across the border.*

### Implied access to authorizations for cross border health

#### Guidance on interstate collaborations in emergencies

Although, there were no explicit rights, entitlements and obligations on cross border healthcare access, we noted some implied access to healthcare across borders in some texts for international treaties. For example, some guidelines addressed physical border crossing, and interstate collaborations during disease surveillance. One policy maker remarked:

*“I know the animal people are more advanced on these issues but the guidelines that we follow are mainly on disease surveillance which is typically about exchange of disease related data across borders” *Policy maker 2, ECSA HQ.

*“ …there is a framework for cross boarder disease surveillance and response which was included in the protocol for regional cooperation in health of EAC. It is a legal framework to ease collaboration among EA countries whenever there is an outbreak on one side of the border or a disease situation that needs intervention of either country” *Policy maker 1, ECSA HQ.

Related to the existing cross border health efforts especially around disease surveillance in the region, stakeholders engaged in the dialogues encouraged efforts to build on the episodic collaboration and projects for HIV, TB and epidemics that usually allow joint collaboration and sharing of resources and investments in the border communities. It was noted these have ad hoc, made remarkable accomplishments but not sustained.

#### Guidelines that promote travel across countries

The regional level (East Africa) documents reviewed provided for East African Integration and promotion of free cross border movements within the region. For example, the treaty for the establishment of the East African Community compels partner states to ease border crossing by citizens of partner states by effecting reciprocal opening of border posts and keeping the posts opened and manned for 24 h. The regional documents also alluded to the need for member states to maintain common standard travel documents for their citizens (i.e. the treaty for the establishment of the EAC. The use of standard travel documents was aimed at achieving free movement of persons within the EAC member states. Considering that travel is one of the requirements for cross border health care access, facilitating border crossing or movements to some extent encourage cross border healthcare access.

From the dialogues, stakeholders highlighted conflicts between states as a major setback for travel across EAC states. They mentioned that b roader political conflicts among states can also pose greater barriers to health care access. During the course of this study, temporary decline in bilateral political relations between Uganda and Rwanda were ongoing [18]. Respondents reported a major decline in service utilization especially on the Uganda side as a result of a stricter border lockdown measures arising from the conflict.

*“The ongoing drama between Uganda and Rwanda government that led to the restriction of movements across our border [Gatuna-Katuna border post] has greatly influenced client attendance at our border health facility [Kamuganguzi HC III] because many of our clients especially on Antiretroviral Therapy (ART) were from the neighboring villages on Rwanda side”*. Health Manager, Kabale stakeholders’ dialogue.

### Institutional frameworks and arrangements

#### Tools that influence or hinder access to healthcare

From the regional and national documents, we noted that some formal arrangements in countries act as tools to facilitate or hinder informal access to health care by cross border communities. The tools included; national identity cards, passports, Rwanda’s Laissez-Passer, yellow fever card among others. There were also arrangements noted such as the Rwanda Community Based Health Insurance Policy which aimed at ensuring universal and equitable access to quality health services to population of Rwanda. However, the CBHI covers only Rwandan Citizens and did not give provision for non-citizens. As such, the Rwandan CBHI and related access rules and cards restricted informal health care access by border resident communities from neighboring countries.

Similarly, the policy makers noted that some formal arrangements that exist in the EAC region facilitated or restricted informal health care access by cross border residents. The policy makers noted that countries with health insurance arrangements for example did not only restrict citizens from the neighboring countries to informally access health care in their countries but were also create financial hardships to their poor citizens unable to enroll or pay premiums. Many residents at the borders were said to have no insurance. On the other hand, countries with free care policy facilitated informal cross border health care access by individuals from the neighboring countries. The policy makers also noted that the need to present referral forms and previous treatment records at the health facilities also hindered formal access to health care by border residents form neighbouring countries.

*In Rwanda the services are available with insurance and can be accessed with a card, but there are many people who don’t have a card especially the vulnerable groups the poor who are close to the border areas. And what they do, they seek all the services from Uganda so on the opposite side you will register all cases because […] you can cross easily without any hindrance. *Policy maker 4, EAC Secretariat.

*“You find that there are a number of reasons that could influence why they cross to the other side. One of them is free services. Uganda is one of the countries in the region which offer free health services. So, people will cross over to Uganda to look for free services and go back to their country”. *Policy maker 3, EAC Secretariat

*One of the policies that actually hinder access is the adamant request for […] referral forms and the need to use previous treatment notes/records. That also I think hinders access to services. *Policy maker 3, EAC Secretariat

### Opportunities for cross border health care access

The regional documents reviewed showed opportunities that could be harnessed to improve cross border health care access. One of the opportunities that can be used to improve cross border health care access is the “Multi-National East African Community Regional Centres of Excellence (CoE) for Skills and Tertiary Education in Higher Medical and Health Sciences Education, Research and Health Services Program” (EASTECO 2018) [19]. According to this program supported by a infrastructure grant from Africa Development Bank, Kenya is earmarked to host the East African Kidney Institute, while the East African Oncology Institute will be located in Uganda. Tanzania will host the East African Heart Institute, while the East African Biomedical Engineering and Health Rehabilitation Institute will be established in Rwanda. These centers are aimed to enable the EAC increase its capacity and competitiveness by expanding top-end higher education and specialized medical services. Although these centers of excellences are for high level care and less for cross border health care access, this futuristic policy explicitly allows people in the EAC to seek health care from member states irrespective of citizenship.

**Fig. 2:**
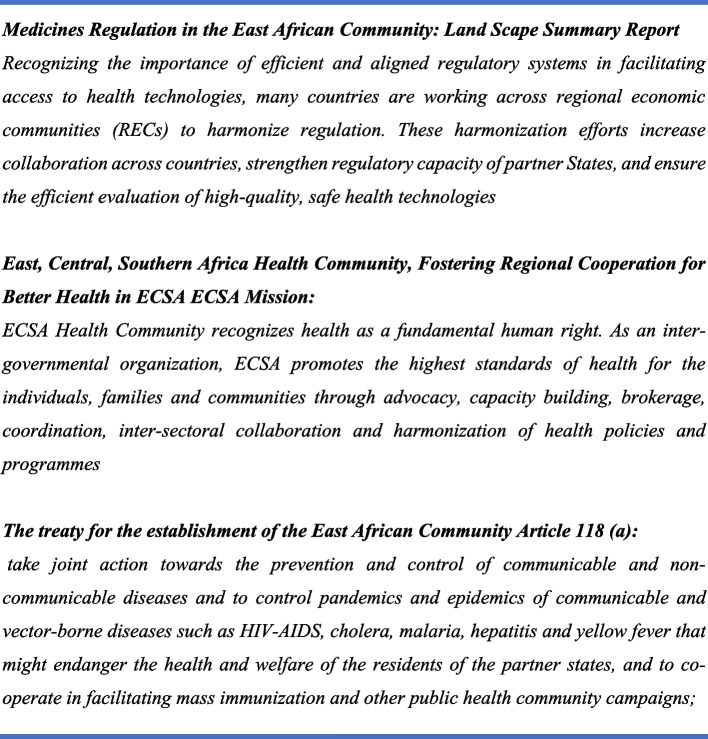
.

Another opportunity that could be sieved to improve cross border health care was the existence of regional bodies such as East Central and Southern Africa (ECSA) Health Community and EAC that promote cross border collaboration among member states. ECSA and EAC promoted cross border collaboration among member states. For instance, The ECSA Health Community has been working with countries and partners to raise the standard of health for the people of the ECSA region by promoting efficiency and effectiveness of health services through cooperation, collaboration, research, capacity building, policy development and advocacy (see Fig. 2).

Relatedly, dialogues by officials responsible for health, migration and related program staff were unanimous about the need to revise policy tools and guidelines to sustain cross-border collaboration, and enable communities to access services where they are available without discrimination. Dialogue participants raised several operational issues to improve. These included mostly the following:

1. There was a need to take care of synergies in health investments to benefit from economies of scale and avoid wasteful and duplicative investments to each side of the border separately. It was noted that hospitals investments were costly and if these are available and in close distance on one side, laws and guidelines should make this accessible by communities on both sides of the border.
2. The twine issue of stigma and adherence was attributed to cross border health access. To ensure their privacy, many cases of HIV and TB were said to seek care across the border. This practice makes the home health system miss targets or report high default rates for chronic care. The need to share data and have joint coordination meetings by neighboring district officials was proposed.
3. Subnational governments especially were decentralization has taken place should be encouraged to negotiate for their communities to enable cross-border planning and resource sharing especially were this is vital to enable sub-national operationalization of the UHC agenda. At the Uganda-Kenya (Busia) border, dialogue meeting observed the opportunity arising from the decentralized administrative zones in both countries and a need to empower them with laws and resources to plan and finance cross border cooperation for human and animal health.

## Discussion

This study aimed to explore the current legal and policy frameworks and how these facilitated or constrained access to healthcare services for communities residing along the East Africa Community (EAC) borders. Findings from this study show that the existing legal and policy frameworks at the Regional and National levels are silent about health care access by communities that reside in border regions of the EAC. Although some legal documents talked about physical movements across the borders, they were not explicit on the movement for seeking health care services. This could be partly because health integration in EAC was still in its infancy stage as compared to economic cooperation and thus, harmonization of policies and guidelines in favor of healthcare access had not taken place. The silence about health care access by communities in border regions is also evident in the Africa Union Free Movement Protocol which talks about free movement of border residents, but it is not explicit on movement for health care or other services for social protection [15]. The literature review found that most of these documents were developed for national-level conventions and purposes and not primarily to guide how social services including health care can be accessed in the advent of the East African Community as a block. This is contrary to the developed world, where explicit guidelines on cross-border health in regional blocks exist. For instance, the European Union (EU), as a part of its regional integration policy, allows equal access to local health facilities for any member state and encourages the exchange of patients between border towns [20]. This explicitness enables the patients in the European Union to exercise their rights to health care even in another country thus ensuring interstate access to health care services.

The international documents reviewed alluded to the right to health and health care, although the enjoyment of these rights and related entitlements were restricted to national-level citizenship. This therefore implies that cross border movements for healthcare access by cross border communities are likewise restricted by State laws. This is also true with healthcare delivery. This finding is also highlighted by the lack of harmonized policies and procedures relating to COVID-19 in East Africa, which limited cross-border cooperation for COVID-19 response in the region [21]. For instance, Uganda charged truckers for COVID-19 testing, while Kenya did not charge, and in Tanzania, testing or wearing of masks was not emphasized. The restriction of the enjoyment of the health and healthcare rights and related entitlements is also observed in Southeast Asia, where the health systems of the Association of Southeast Asian Nations (ASEAN) are limited to within the borderline of each member state [20]. In particular, the right to health care is only granted to citizens or documented transnational migrants who live and work legally.

This study noted tools for example; health insurance, travel documents among others, that influenced healthcare access for border resident communities. For example, East African countries that had health insurance cover for their citizens tended to restrict access to healthcare by border residents from neighbouring countries since they were not covered. This is contrary to elsewhere in the developed world, where cross-border health is advanced and there provisions for health insurance across borders. For example, a study by Miller-Thayer (2010) showed that US border residents could obtain care throughout the entire year in Mexico, despite the presence of health insurance in Mexico [3]. The above was facilitated by the presence of border insurance policies that covered the healthcare border residents receive in Mexico. Still regarding tools, a study in West Africa by Opanike and Aduloju (2015) reported tools that restricted cross-border movements. The study reported that cross border movements were increasingly hampered by migration and custom laws as well as tools and requirements that these entail [22].

### Program and policy implications

This study highlights several program and policy implications. Firstly, the lack of explicit policy on cross-border healthcare access especially for border residents highlights the urgent need for EAC member states to consider developing an explicit policy on cross-border healthcare in the region in order to improve accessibility of healthcare services in cross border regions of East Africa community and neighboring countries. This could be similar to the European Union Directive on cross-border healthcare [20] and could help define the scope of cross border health services and their coverage by national healthcare systems. Secondly, the EAC member States could cede authority to the sub-national administrative zones to enable greater cross-border healthcare cooperation and coordination to serve communities on both side of their borders by optimizing the health care investments in these communities. Good communication and mutual understanding between national programmes that share a border are arguably the most important keys to ensuring that Cross Border situations do not impede the progress to elimination [23]. Thirdly, there is also need to have the current treatment protocols harmonized across all EAC member States in order to facilitate uniformity and continuity of care. Relatedly, the variations in some of the tools that facilitate healthcare access i.e. health insurance, and travel documents, should be harmonize where possible, and put in place special considerations for populations that reside in the border regions. Payments systems and price transfer systems should be explored to enable decentralized administrative units to plan, negotiate and finance the health care needs that get served across the border. Lastly, this study also noted ongoing efforts for regionalization of advanced health care services like cancer care, kidney disease and dialysis, heart disease and others similar services, however many of these were still at the planning stage with nonexistent legal framework to guide how these centers will be financed, and accessed by citizens from the EAC member states. This calls for an expedited revision of the legal and institutional frameworks to enable health care planning, financing and access for both the border communities and the East African community as a block.

### Strength and limitations

This paper contributes to an emerging literature on cross border healthcare by bringing into focus one element that has been hidden in these debates – vulnerability of non-citizens in accessing healthcare and the need to extend essential social protection services and entitlements for non-citizens residing along national borders. The paper highlights that the issue of cross-border healthcare should be an inter-sectoral agenda and not limited within the health sector as it involved, e.g., immigration/ security.

We used multiple data sources i.e., document review, Key informant interviews, and dialogue meetings to develop a comprehensive understanding of the phenomena hence data source triangulation.

Important to note are the limitations of this study. The study focused on only three countries in the EAC. Despite the limitation, we believe the study presents important insights into how legal and institutional frameworks constrain or facilitate access to healthcare for residents in border areas in the study countries, hence informative for the broader EAC integration agenda.

## Conclusion

Findings from this study highlight three important needs for cross-border healthcare to take course in EAC; (1) creation of a supportive policy environment by aligning the existing policies, and or formulating explicit policies on cross-border healthcare in EAC; (2) making investments in regionalization of healthcare or national collaborations in health. Key gaps and dysfunctions identified in this study can be used to inform regional and national discussions on cross border healthcare planning, financing and access among policy makers and implementers, and (3) there is urgent need for smart ways to invest in improving access to health and other social goals like UHC and SDG. Cross border cooperation to expand access and mitigate duplication will require a legal and institutional framework that welcomes cooperation among EAC member states and affirmative policies and investments that extents rights and entitlements to the citizenship of the EAC block with particular emphases given to remote communities along the borders within the EAC.

### Ethical considerations

The study obtained approval from the higher degrees, research and ethics committee of the School of Public Health, Makerere University (HDREC Protocol number 583) and the Uganda National Council for Science and Technology under study number SS4713. For the Key Informant interviews, we obtained written consent from all participants after explaining the purpose of the study. The respondents were also informed about the voluntary nature of the study and confidentiality was assured throughout the interviews.

## Supplementary Information

**Additional file 1.** Cross Border Health Access Study_Key Informant Guide.

## Data Availability

Data are available on reasonable request. The data that support the findings of this study are available on request from the corresponding author SB.
