# Supplementary material for: The right of access to healthcare: an analysis of how legal and institutional frameworks constrain or facilitate access to healthcare for residents in border areas in the East African Community
Source: Int J Equity Health. 2022 Nov 26;21:168. doi: 10.1186/s12939-022-01785-3 (PMC9701445; doi:10.1186/s12939-022-01785-3)
Supplement: Supplementary file 1 — Additional file 1. Cross Border Health Access Study_Key Informant Guide. [file 12939_2022_1785_MOESM1_ESM.pdf]

Cross Border Health Access Study_24-05-018

Legal and institutional barriers to healthcare access for resident border communities

KI Guide for regional and national level stakeholders

This interview should be completed with; **EAC representatives, ECSA Country representatives, National ministerial representatives of Health and International Affairs.**<br><br>**Note:** *Once consent forms are signed, begin recording the interview. Use the space after each question to enter notes.*	
F1. **Date**: Day: [ ][ ] Month: [ ][ ] ear: [ ][ ]	**F3.** Interviewee details:<br>Age: ______________________<br>Position: ____________________<br>Years in the position: _________
F2. Interviewee ID:	F4. Country name/ Cross Border Site<br><br>____

Note to Interviewer: Key Definitions

For this assessment;

- ***Cross boarder resident community*** is defined as a group of people who live on boarders
- *Access* is defined as entry into or use of the health care system
- ***Health services*** are define as a service that provides medical treatment and care to the public or to a particular group
- ***An enabler to access*** is defined as a factor both personal and/or environmental that facilitates a client to seek and use health services from across the boarder
- ***A barrier to access*** is defined as a factor both personal and/ or environmental that hinders a client from seeking and using health services from across the boarder

**AVAILABILITY AND USE OF HEALTH SERVICES**<br><br>1. As a country representative, comment about how the populations residing at national boarders access health services?

2. What arrangements are in place to promote, protect and fulfill health access rights for people residing on either side of national borders (10 Km radius along the border line)?

a) When planning for service delivery, do you put provisions to enable people who reside along international borders to freely access health services? *Probe for structures, policies and resources*

b) Within service delivery, what provisions restrict access to health services for people who reside along international borders to freely access health services? *Probe for structures, policies and resources*

INSTITUTIONAL FRAMEWORKS FOR CROSS BORDER HEALTH CARE ACCESS IN YOUR COUNTRY

3. At regional or country level, what guidelines are there for allowing or not allowing people to cross and use health services in the neighboring countries? *Probe for documents, procedure, course of actions*

4. In a situation when someone from the opposite side of the national border is sick, what considerations or requirements do you have to allow or not allow them to access health services in your country?

a) Under what situations, do you allow or not allow children under five years from the opposite side of the national borders to receive immunization services in your country?

b) Under what situations, do you allow or not allow a pregnant woman from the opposite side of the national borders to receive caesarian section services (EmMOC) in your country?

c) Under what situations, do you allow or not allow persons from the opposite side of the national borders to receive HIV care services for treatment refill in your country?

5. Are there considerations that have to be emphasized to allow persons from the opposite side of the national borders to access health services in your country?

6. Are there considerations that can be waved or over looked for some reason to allow the person from the opposite side to access health services in your country?

FACILITATORS AND BARRIERS TO HEALTH CARE SERVICESS ACCESS BY RESIDENT BOARDER

COMMUNITIES

7. In your capacity as a country-level representative, tell us what enables people from the opposite side of the national borders to access health services from your country? ***Probe for structures, policies and resources***

8. In your capacity as a country-level representative, tell us what hinders people from the opposite side of the national borders to access health services from your country? ***Probe for structures, policies and resources***
